# Differential activation of human core, non-core and auditory-related cortex during speech categorization tasks as revealed by intracranial recordings

**DOI:** 10.3389/fnins.2014.00240

**Published:** 2014-08-11

**Authors:** Mitchell Steinschneider, Kirill V. Nourski, Ariane E. Rhone, Hiroto Kawasaki, Hiroyuki Oya, Matthew A. Howard

**Affiliations:** ^1^Departments of Neurology and Neuroscience, Albert Einstein College of MedicineBronx, NY, USA; ^2^Human Brain Research Laboratory, Department of Neurosurgery, The University of IowaIowa City, IA, USA

**Keywords:** electrocorticography, Heschl's gyrus, high gamma, prefrontal cortex, semantics, speech, superior temporal gyrus

## Abstract

Speech perception requires that sounds be transformed into speech-related objects with lexical and semantic meaning. It is unclear at what level in the auditory pathways this transformation emerges. Primary auditory cortex has been implicated in both representation of acoustic sound attributes and sound objects. While non-primary auditory cortex located on the posterolateral superior temporal gyrus (PLST) is clearly involved in acoustic-to-phonetic pre-lexical representations, it is unclear what role this region plays in auditory object formation. Additional data support the importance of prefrontal cortex in the formation of auditory objects, while other data would implicate this region in auditory object selection. To help clarify the respective roles of auditory and auditory-related cortex in the formation and selection of auditory objects, we examined high gamma activity simultaneously recorded directly from Heschl's gyrus (HG), PLST and prefrontal cortex, while subjects performed auditory semantic detection tasks. Subjects were patients undergoing evaluation for treatment of medically intractable epilepsy. We found that activity in posteromedial HG and early activity on PLST was robust to sound stimuli regardless of their context, and minimally modulated by tasks. Later activity on PLST could be strongly modulated by semantic context, but not by behavioral performance. Activity within prefrontal cortex also was related to semantic context, and did co-vary with behavior. We propose that activity in posteromedial HG and early activity on PLST primarily reflect the representation of spectrotemporal sound attributes. Later activity on PLST represents a pre-lexical processing stage and is an intermediate step in the formation of word objects. Activity in prefrontal cortex appears directly involved in word object selection. The roles of other auditory and auditory-related cortical areas in the formation of word objects remain to be explored.

## Introduction

Speech perception requires that incoming sounds be transformed into word objects. It is unclear at what level in the auditory pathways this transformation occurs. Some data suggest that primary auditory cortex principally represents acoustic sound attributes (Mesgarani et al., 2008; Poeppel et al., 2008; Steinschneider et al., 2013). Other data suggest that primary auditory cortex is more directly involved in sound object representation (Nelken, 2008; Nelken and Bar-Yosef, 2008). It is also unclear what role non-primary auditory cortex, located on the posterolateral superior temporal gyrus (PLST), plays in object formation. PLST is critical for acoustic-to-phonetic transformations (Boatman, 2004; Poeppel et al., 2008; Chang et al., 2010; Steinschneider et al., 2011; Mesgarani et al., 2014). This process could be interpreted as a remapping of the speech signal from one encoding acoustic attributes to one representing its phonemic components. By extension, it could be argued that this process remains a precursor to the formation of word objects. In this scheme, word object formation would be expected to take place at higher levels in auditory and auditory-related cortex (Griffiths and Warren, 2004; Griffiths et al., 2012).

Multiple studies have examined the transformation of neural activity associated with the representation of sound attributes to a representation of sound objects (Griffiths and Warren, 2004; Winkler et al., 2006; Shinn-Cunningham, 2008; Alain and Winkler, 2012; Griffiths et al., 2012; Simon, 2014). At the object formation processing stage, neural activity associated with a specific object must be distinct from that associated with other sound objects. Further, the neural representation of an object must be relatively invariant to variations in the detailed acoustics of the sounds. For instance, the representation of a specific word and its meaning must remain stable despite variations in acoustic characteristics that occur when a given word is spoken by different talkers. Given these requirements, object formation can be evaluated by utilizing tasks that require classifying words into semantic categories (Shahin et al., 2006; Hon et al., 2009).

Intracranial electrophysiological recordings in humans offer a unique opportunity for studying task-related activity in auditory cortex that accompanies semantic processing of speech. The technique combines exquisite spatial and temporal resolution beyond that offered by non-invasive methods such as neuromagnetic responses and functional magnetic resonance imaging (MRI) (e.g., Lachaux et al., 2012). An excellent example of the sensitivity and specificity provided by intracranial recordings in humans is the study demonstrating that competing speech signals can be segregated according to speaker through analysis of cortical activity recorded from PLST during selective attention tasks (Mesgarani and Chang, 2012). The neural activity associated with the attended stream was enhanced, while activity associated with the unattended stream was suppressed. In a related study, target detection tasks led to enhanced neural activity to target tone stimuli on PLST when compared to responses obtained during passive listening and responses to non-target tone stimuli (Nourski et al., 2014a). These effects occurred during later portions of the neural responses. Early activity was minimally affected by the task requirement and appeared to represent the acoustic attributes of the tones. Similarly, minimal effects were noted in activity simultaneously recorded from posteromedial Heschl's gyrus (HG), the putative location of core auditory cortex. These findings suggest that activity generated within posteromedial HG and early activity from PLST reflect acoustic encoding rather than the representation of non-speech and speech-related objects at the phonemic level. It remains unclear from these studies, however, if this region of auditory cortex will also be involved in the formation of speech-related objects at the level of words and their semantic meaning.

The current study focused on high gamma responses (70–150 Hz) generated during target detection tasks using both speech and non-speech stimuli. High gamma activity has been shown to be a sensitive and specific indicator of auditory cortical activation and has been successfully used to define organizational features of human auditory cortex (e.g., Crone et al., 2001; Steinschneider et al., 2008, 2011; Flinker et al., 2010; Mesgarani and Chang, 2012; Mesgarani et al., 2014; Nourski et al., 2014b). Tasks of the current study included detecting words belonging to specific semantic categories or talker gender, as well as the detection of tones intermixed with the word sequences. Words were consonant-vowel-consonant exemplars from the semantic categories of animals, numbers and colors, as well as nonsense syllables, each spoken by different male and female talkers. Therefore, neural activity associated with target detection should not be based solely on acoustic attributes and instead should be related to semantic categorization and, consequently, word object formation. We predicted that the tone detection task would not engage speech-related object formation, as this task only required differentiating the sound objects based on their acoustic attributes. In contrast, tasks that required the subject to detect words from a specific target category necessitated that words be decoded and categorized as sound objects belonging to specific semantic categories. Detection of talker gender provided an intermediate control condition. If the successful completion of the task was solely dependent upon decoding the fundamental frequencies typically encountered across gender (e.g., Hillenbrand et al., 1995), then, we hypothesized, sound object formation would not engage word-specific processing. If, however, formation of word objects incorporated representation of gender, then response profiles should be similar to that observed when words were categorized along semantic categories.

We also examined neural activity within auditory-related cortical areas that have been shown to be critical components of the neural network subserving speech perception (e.g., Rauschecker and Scott, 2009). Neural activity from inferior frontal gyrus (IFG) in the language-dominant hemisphere measured with intracranial recordings has been shown to represent lexical, grammatical and phonological aspects of speech (e.g., Sahin et al., 2009). In the present study, responses from the portion of IFG that overlaps with classically defined Broca's area were compared with activity recorded from HG and PLST. Additionally, contributions from middle temporal gyrus (MTG) and middle frontal gyrus (MFG) were examined, as these higher-order cortical regions may also be involved in word object formation (Griffiths and Warren, 2004; Poeppel et al., 2008). Simultaneous recordings from multiple regions including core, non-core and auditory-related cortex provided a unique opportunity to examine the role of each of these areas in word object formation during target detection tasks with high temporal and spatial detail.

## Methods

### Subjects

Experimental subjects were three neurosurgical patients diagnosed with medically refractory epilepsy and undergoing chronic invasive electrocorticographic (ECoG) monitoring to identify potentially resectable seizure foci. The subjects were 38 (L258), 30 (L275), and 40 (L282) years old. All subjects were male, right-handed and left hemisphere language-dominant, as determined by intracarotid amytal (Wada) test results. Recordings were obtained from the left hemisphere in all three subjects. Research protocols were approved by the University of Iowa Institutional Review Board and the National Institutes of Health. Written informed consent was obtained from all subjects. Research participation did not interfere with acquisition of clinically required data, and subjects could rescind consent at any time without interrupting their clinical evaluation.

All subjects underwent audiometric evaluation before the study, and none was found to have hearing deficits that should impact the findings presented in this study. Subjects L258 and L282 were native English speakers, and subject L275 was a native Bosnian speaker who learned German at the age of 10 and English at the age of 17. Neuropsychological testing of L258 was normal except for mild deficiencies in verbal working memory. Subject L275 had grossly intact conversational language comprehension, though formal neuropsychological testing showed non-localizing cognitive function deficits. Subject L282 had 13 years earlier undergone anterior temporal lobectomy that spared auditory cortex on the superior temporal gyrus. This subject was found to have mild deficits in verbal memory, fluency and naming. However, all three subjects had comparable performance in all experimental tasks both in terms of target detection accuracy and reaction times. This indicates that their performance of the tasks was not limited by any cognitive deficits identified during formal neuropsychological testing. Intracranial recordings revealed that auditory cortical areas were not epileptic foci in any subject.

Experiments were carried out in a dedicated electrically-shielded suite in The University of Iowa General Clinical Research Center. The room was quiet, with lights dimmed. Subjects were awake and reclining in an armchair.

### Stimuli

Experimental stimuli were consonant-vowel-consonant syllables [cat], [dog], [five], [ten], [red], [white], [res], and [tem] from TIMIT (Garofolo et al., 1993) and LibriVox (http://librivox.org/) databases. Non-word syllables were excised from words using SoundForge 4.5 (Sonic Foundry Inc., Madison, WI). A total of 20 unique exemplars of each syllable were used in each experiment: 14 spoken by different male and 6 by different female speakers. Additionally, the stimulus set included complex tones with fundamental frequencies of 125 (28 trials) and 250 Hz (12 trials), approximating the average voice fundamental frequencies of male and female speakers, respectively. All stimuli were normalized to the same root-mean-square amplitude and edited to be 300 ms in duration using SoundForge with 5 ms rise-fall times. They were presented with an inter-stimulus interval chosen randomly within a Gaussian distribution (mean interval 2 s; *SD* = 10 ms) to reduce heterodyning in the recordings secondary to power line noise. Stimuli were delivered via insert earphones (ER4B, Etymotic Research, Elk Grove Village, IL) that were integrated into custom-fit earmolds. Stimulus delivery was controlled using Presentation software (Version 16.5 Neurobehavioral Systems, http://www.neurobs.com/).

The same stimuli were presented in random order in multiple target detection tasks. The target stimuli were either complex tones (presented as first block in each subject), speech stimuli spoken by female talkers, or words belonging to specific semantic categories such as animals or numbers. The subjects were instructed to use the index finger of their left hand (ipsilateral to the recording hemisphere) to push the response button whenever they heard a target sound. Prior to data collection, the subjects were presented with a random-sequence preview of stimuli to ensure that the sounds were presented at a comfortable level and that they understood the task requirements.

### Recordings

ECoG recordings were simultaneously made from HG and lateral cortical surface using multicontact depth and subdural grid electrodes, respectively. Details of electrode implantation have been described previously, and more comprehensive details regarding recording, extraction and analysis of high gamma cortical activity are available for the interested reader (Howard et al., 1996, 2000; Reddy et al., 2010; Nourski et al., 2013; Nourski and Howard, 2014). In brief, hybrid depth electrode arrays were implanted stereotactically into HG, along its anterolateral to posteromedial axis. In subject L258, a hybrid depth electrode was used, which contained 4 cylindrical platinum macro-contacts, spaced 10 mm apart, and 14 platinum micro-contacts, distributed at 2–4 mm intervals between the macro contacts. In subjects L275 and L282, a depth electrode with 8 macro-contacts, spaced 5 mm apart, was used. Subdural grid arrays were implanted over the lateral surface of temporal and frontal lobes in subjects L258 and L275. The grid arrays consisted of platinum-iridium disc electrodes (2.3 mm exposed diameter, 5 mm center-to-center inter-electrode distance) embedded in a silicon membrane. The electrodes were arranged in an 8 × 12 grid, yielding a 3.5 × 5.5 cm array of 96 contacts. A subgaleal contact was used as a reference. Electrode arrays were placed solely on the basis of clinical requirements, and were part of a more extensive set of recording arrays meant to identify seizure foci. Electrodes remained in place under the direction of the patients' treating neurologists.

Subjects underwent whole-brain high-resolution T1-weighted structural MRI scans (resolution 0.78 × 0.78 mm, slice thickness 1.0 mm) before electrode implantation to locate recording contacts. Two volumes were averaged to improve the signal-to-noise ratio of the MRI data sets and minimize the effects of movement artifact on image quality. Pre-implantation MRIs and post-implantation thin-sliced volumetric computerized tomography scans (resolution 0.51 × 0.51 mm, slice thickness 1.0 mm) were co-registered using a linear co-registration algorithm with six degrees of freedom (Jenkinson et al., 2002). Locations of recording sites were confirmed by co-registration of pre- and post-implantation structural imaging and aided by intraoperative photographs.

Data acquisition was controlled by a TDT RZ2 real-time processor (Tucker–Davis Technologies, Alachua, FL). Collected ECoG data were amplified, filtered (0.7–800 Hz bandpass, 12 dB/octave rolloff), digitized at a sampling rate of 2034.5 Hz, and stored for subsequent offline analysis. Behavioral responses to the target stimuli were recorded using a Microsoft SideWinder game controller. The timing of the button-press events was recorded and stored for analysis along with ECoG data.

### Data analysis

ECoG data obtained from each recording site were downsampled to a rate of 1000 Hz. To minimize contamination with power line noise, ECoG waveforms were de-noised using an adaptive notch filtering procedure (Nourski et al., 2013). Prior to calculation of high gamma event-related band power (ERBP), individual trials were screened for possible contamination from electrical interference, epileptiform spikes, high amplitude slow wave activity, or movement artifacts. To that end, individual trial waveforms with voltage exceeding 2.5 standard deviations from the mean were rejected from further analysis. Data analysis was performed using custom software written in MATLAB Version 7.14 programming environment (MathWorks, Natick, MA, USA).

Quantitative analysis of the ERBP focused on the high gamma ECoG frequency band. High gamma ERBP was calculated for each recording site. Single-trial ECoG waveforms were bandpass filtered between 70 and 150 Hz (100th order finite impulse response filter) and squared. The resultant high gamma power waveforms were smoothed using a moving average filter with a span of 25 ms, log-transformed, normalized to power in a pre-stimulus reference (250-50 ms prior to stimulus onset), and averaged across trials. To assess the presence and timing of task-related modulation of high gamma activity on representative cortical sites, single-trial high gamma ERBP was first averaged in 50 ms-wide consecutive windows to decrease the number of multiple comparisons. Next, for each window from 0–50 to 950–1000 ms, a two-sample one-tailed *t*-test was performed on single-trial windowed ERBP values to compare responses to stimuli presented in the non-target (tones task) and target condition. Finally, *p*-values were corrected for multiple comparisons (i.e., recording sites and time windows) using false discovery rate by controlling the false discovery rate following the method of Benjamini and Hochberg (1995) and Benjamini et al. (2001) with a threshold of *q* = 0.01.

## Results

### HG

**Figure 1:**
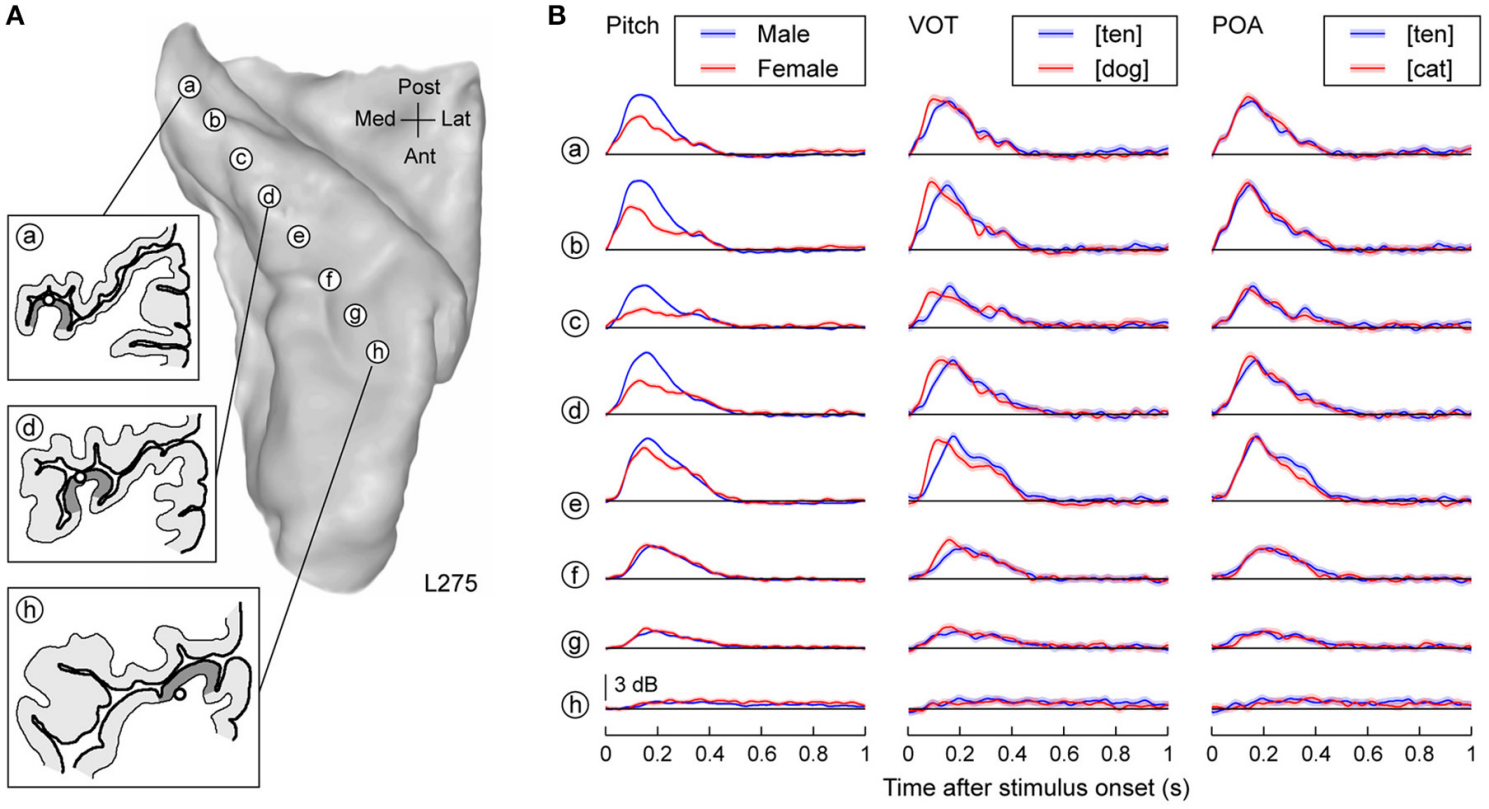
**Representation of acoustic stimulus attributes in HG. (A)** MRI of left superior temporal plane in subject L275 showing the locations of recording contacts chronically implanted in HG. Insets: tracings of MRI cross-sections showing the location of three recording contacts (circles) relative to the gray matter of the HG (dark gray shading). **(B)** High gamma responses to speech sounds differing in pitch, initial stop consonant VOT and POA are shown in the left, middle and right column, respectively. Lines and shaded areas represent mean high gamma ERBP and its standard error, respectively.

Neural activity on HG primarily represented acoustic attributes of the speech stimuli (Figure 1). Figure 1A illustrates the location of the eight recording contacts that targeted HG along its long axis in subject L275. Mean high gamma power elicited by three acoustic attributes of speech is shown for each recording site (Figure 1B). Responses to the speech stimuli spoken by male talkers were consistently larger compared to those elicited by female talkers (Figure 1B, left column), reflecting differences in their fundamental frequency (male talkers: mean 125 Hz, *SD* 25 Hz; female talkers: mean 202 Hz, *SD* 36 Hz). These differences represent a contribution in the high gamma responses of phase locking to the lower fundamental frequency of the male talkers within posteromedial HG [sites (a) through (d)] (cf. Nourski and Brugge, 2011; Steinschneider et al., 2013).

Voice onset time of the initial stop consonants was also differentially represented in the high gamma activity. In general, high gamma activity peaked earlier for initial consonants with short voice onset times (VOTs) (i.e., [dog]) relative to those with more prolonged VOTs (i.e., [ten]). This effect was maximal in more central portions of HG compared to the observed effect of pitch on neural activity [sites (e), (f); Figure 1B, middle column]. Differences based upon initial consonant place of articulation (POA) were more subtle, likely due to the overlap in spectral content across the stimulus exemplars (e.g., site (d); Figure 1B, right column). These patterns of activity within HG were also observed in the other two subjects (Supplementary Figures 1, 2).

**Figure 2:**
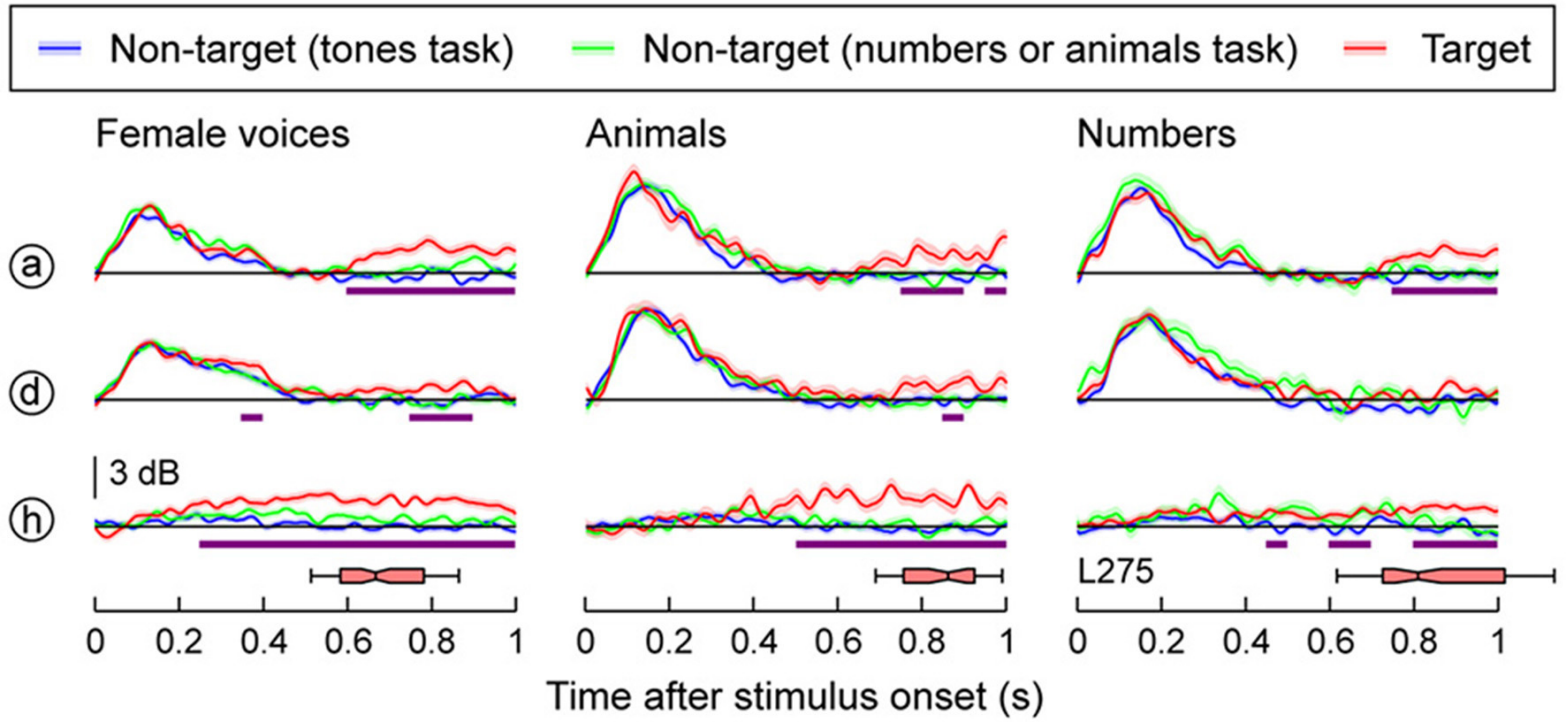
**Task effects on responses to speech stimuli in HG**. Responses to three types of stimuli (female voices, animals, numbers; left, middle and right column, respectively) are shown for three representative recording sites in HG (rows). See Figure 1A for location of the recording sites. Colors (blue, green, and red) represent different task conditions. Lines and shaded areas represent mean high gamma ERBP and its standard error, respectively. Purple bars denote time windows where responses to the target stimuli were significantly larger than those to the same stimuli in the tones task (*q* < 0.01). Horizontal box plots denote the timing of behavioral responses to the target stimuli (medians, 10th, 25th, 75th, and 90th percentiles).

Whereas activity along most of HG was strongly modulated by the acoustic attributes of the sounds, responses in the high gamma range were only weakly affected by the target detection tasks (Figure 2). The left column in Figure 2 compares neural activity to the same set of stimuli (female voices) in three blocks: when they were targets, when they were non-targets in the tone detection block, or when they were non-targets in a semantic task (numbers). A low-amplitude increase in high gamma was seen beginning within 600–650 ms after stimulus onset when female voices were the targets [site (a)], overlapping in time with the subject's behavioral response. A similar effect was seen for responses to the animals and numbers when they were the targets. However, the onset of the task-related high gamma modulation in these semantic categorization conditions was even slower than that occurring during voice identification task (*q* < 0.01 at 750–800 ms after stimulus onset; middle and right columns of Figure 2).

A different pattern was observed within the most anterolateral portion of HG outside of presumed core cortex [site (h) in Figure 2]. Here the response was delayed relative to the activity on posteromedial HG and was specifically associated with target stimuli. Importantly, this task-related activity preceded task-related changes that were observed on posteromedial HG. These task-related increases, however, were variable across subjects. In the other two subjects, no significant task-related effects were observed at the level of either posteromedial or anterolateral HG (Supplementary Figures 3, 4). Thus, in total, task-related changes in HG were, as we will show, modest, when compared to those changes observed on PLST and in auditory-related cortex.

### PLST

**Figure 3:**
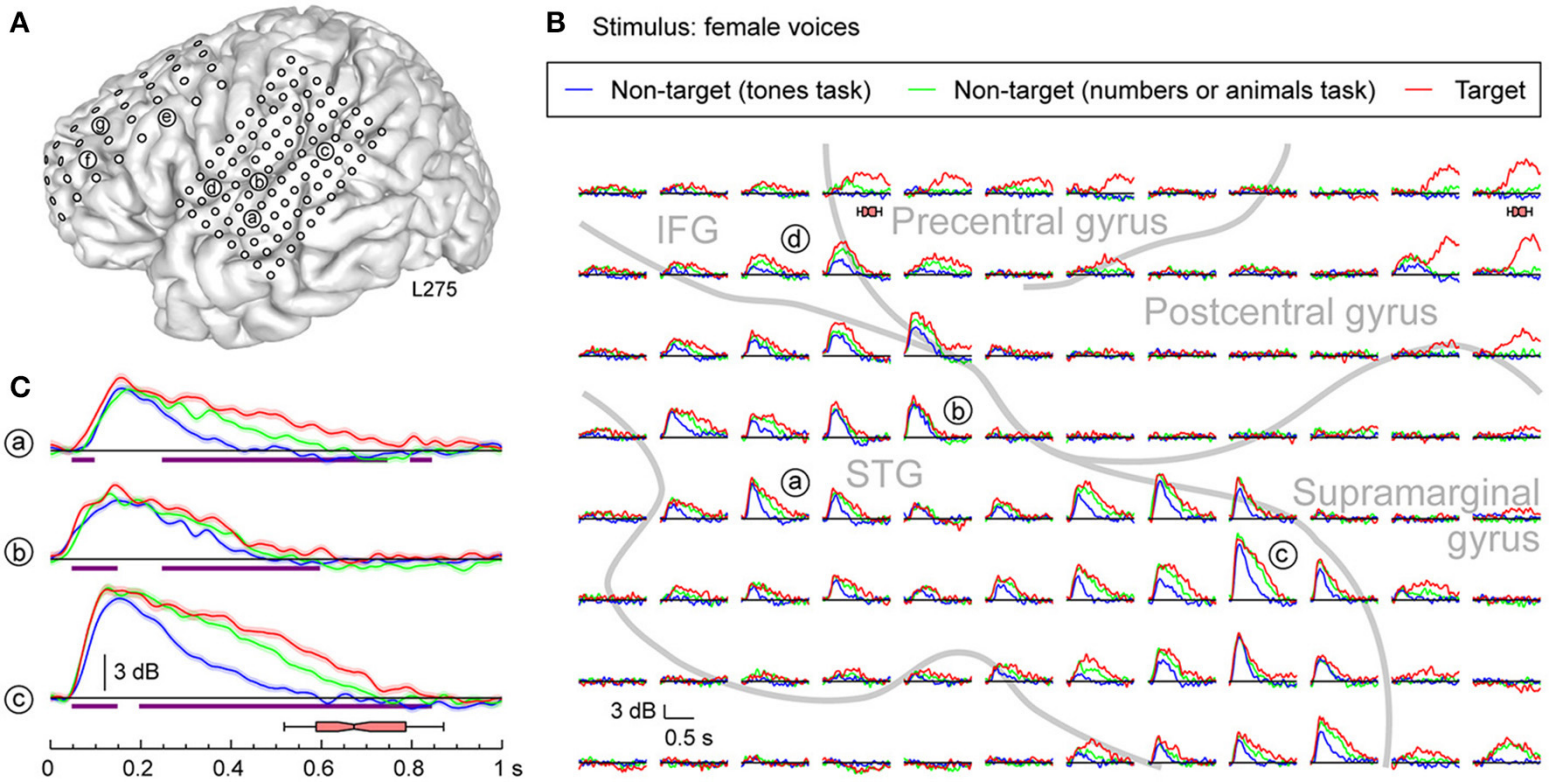
**Task effects on responses to speech stimuli (female voices) in PLST. (A)** MRI of the left hemisphere in subject L275 showing the locations of chronically implanted subdural grid contacts. **(B)** High gamma responses to syllables spoken by females, presented in three different tasks (different colors), are shown for the 96-contact recording grid implanted over perisylvian cortex. Gray lines represent approximate boundaries of STG, IFG, pre- and post-central gyri covered by the recording grid. **(C)** High gamma ERBP time course replotted for three recording sites on PLST. Lines and shaded areas represent mean high gamma ERBP and its standard error, respectively. Purple bars denote time windows where responses to the target stimuli were significantly larger than those to the same stimuli in the tones task (*q* < 0.01). Horizontal box plot denotes the timing of behavioral responses to the target stimulus (median, 10th, 25th, 75th, and 90th percentiles).

**Figure 4:**
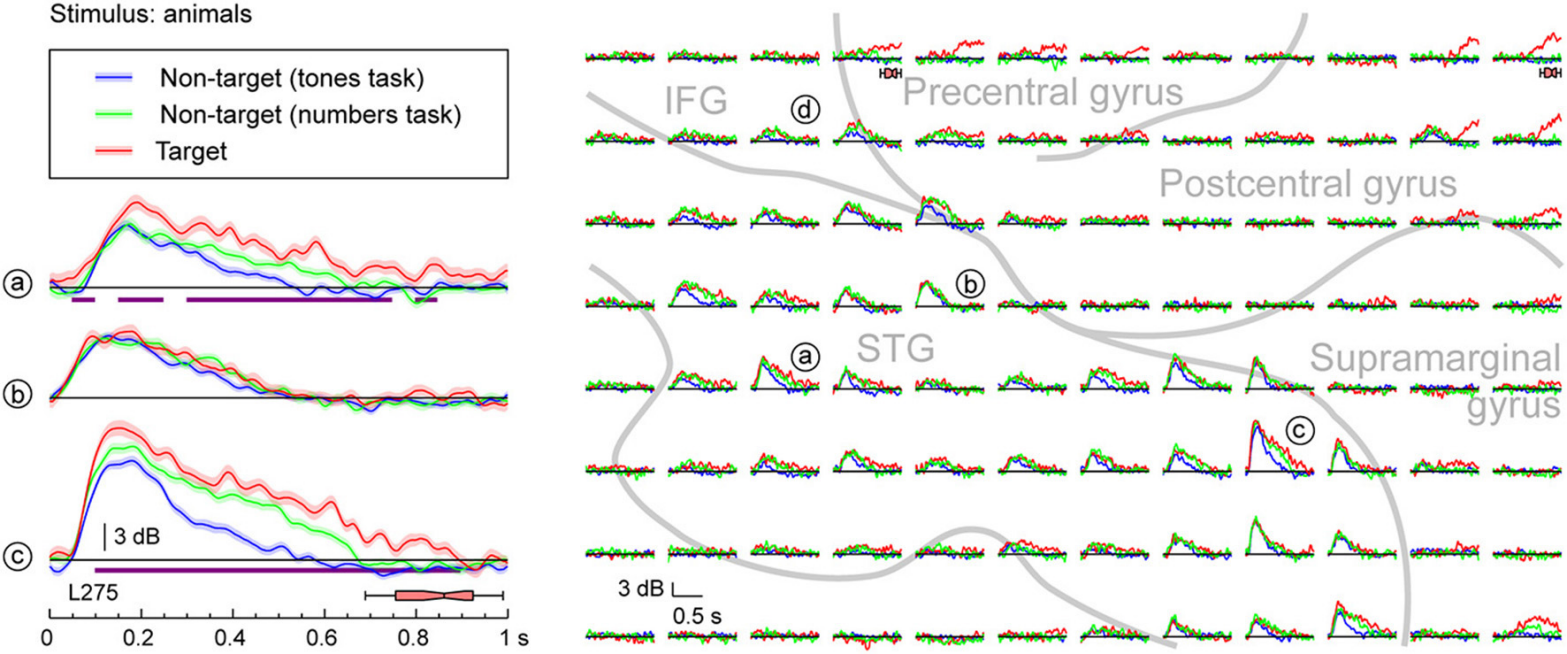
**Task effects on responses to speech stimuli (animals) in PLST**. High gamma responses to speech sounds representing animals, presented in three different tasks (different colors), are shown for three representative contacts and the entire 96-contact recording grid implanted over perisylvian cortex (left and right panels, respectively). See legend of Figure 3 for details and location of recording sites.

More complex response profiles were observed on PLST (Figures 3, 4) when compared with profiles simultaneously recorded from HG (see Figure 2). There was a rapid and large increase in high gamma ERBP occurring within 200 ms after stimulus onset. This early activity was variably affected by the task [e.g. sites (a), (b), and (c) in Figures 3, 4]. When female voices were targets, a modest but significant increase in high gamma power was observed as early as 50–100 ms after stimulus onset. Peak activity at 150–200 ms was only marginally affected by the task. Later activity was more variable across recording sites. Both enhancement of high gamma activity to the target syllables beginning prior to their offsets [e.g. sites (a), (b), and (c) in Figure 3] and minimal modulation of later activity related to the task (see Figure 3B) were observed in this region. Task-related high gamma activity was earlier than that occurring in HG (cf. Figure 2) and preceded the subject's behavioral response.

Responses to non-target words were also modulated by the specific task requirements. For instance, late high gamma activity to non-target words spoken by females was enhanced when the target detection tasks required words to be categorized relative to the task where complex tones were the targets (see Figure 3, green and blue plots, respectively). Responses to female voices when they were target stimuli were consistently larger than when they were non-targets, even though the subject was engaged in cognitively more demanding tasks (detecting numbers or animals) (see Figure 3, red and green plots, respectively). The difference in task difficulty can be inferred from behavioral response times, which were significantly shorter when the target was female voices (median response time 672 ms) relative to either task requiring semantic classification (animals: median response time 866 ms; numbers: median response time 815 ms) (*p* < 0.001, Mann-Whitney rank-sum tests).

Enhancement of high gamma power was also observed when the targets were animals (Figure 4). Once again, targets elicited the largest responses when compared to when they were non-targets presented in a tone detection task [see Figure 4, sites (a) and (c)]. While variable across sites [cf. site (b) in Figure 4], enhanced activity could occur early and remain elevated even during the time period of the behavioral response. Responses to non-target animal words presented in a different semantic categorization task (detecting numbers) were intermediate in magnitude. The behavioral reaction times were comparable in the animals and numbers detection tasks (*p* = 0.71, Mann–Whitney rank-sum test). Therefore, it is reasonable to conclude that these differences between target and relevant non-target were not based solely on task difficulty. Importantly, increases in high gamma activity observed during either semantic categorization task began prior to the offset of the syllables, suggesting that these increases were not directly related to word classification, and likely were reflecting lower-level phonological processing, a prerequisite for semantic classification (cf. Boatman, 2004).

In subject L258, task-related enhancement was not observed from sites located on PLST [Supplementary Figure 5; sites (a), (b), and (c)]. This negative finding may reflect in part differences in placement of the electrode grids, where the anterior limit of the temporal recording grid was anatomically more posterior than that in subject L275. Additionally, responses from L275 were averaged over a larger number of trials, improving signal-to-noise ratio, and subject L275 was generally more enthusiastic about performing the behavioral tasks compared to L258. However, responses were modulated by the task on sites overlying the MTG [e.g. sites (d) and (e); Supplementary Figure 5], similar to that seen on PLST in subject L275. Specifically, late responses to target stimuli were larger than responses in the tone detection task, reaching significance on site (e) in the gender identification task (*q* < 0.01), and were marginally significant (*q* < 0.05) in the semantic categorization tasks on sites (d) and (e) (significance bars are not shown). Additionally, there was a trend for non-target words to elicit larger late responses during semantic categorization tasks compared to tone detection (green and blue plots, respectively, in Supplementary Figure 5).

### Auditory-related cortex: IFG and MFG

**Figure 5:**
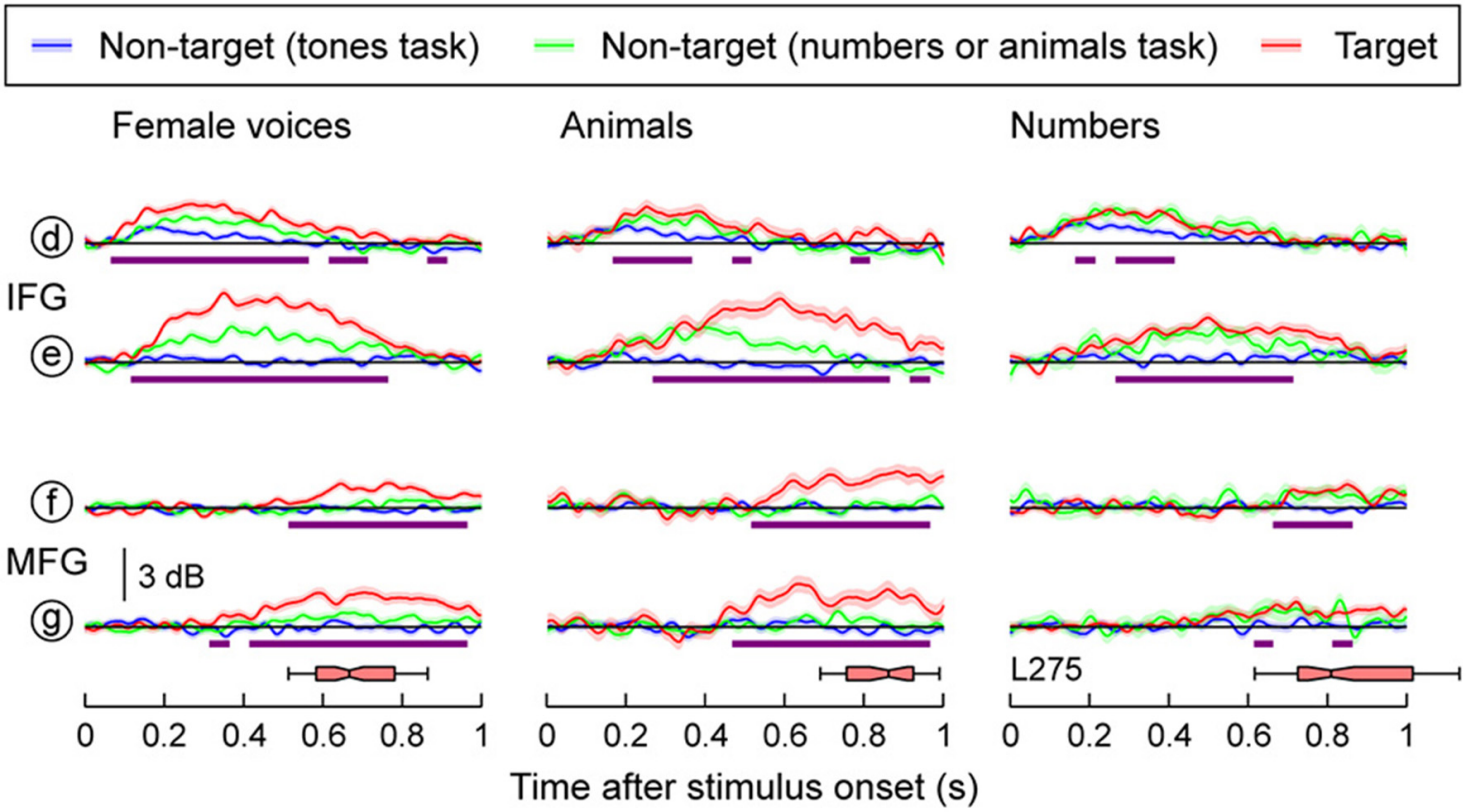
**Task effects on responses to speech stimuli in IFG and MFG**. High gamma responses to three types of stimuli (female voices, animals, numbers; columns), presented in three different tasks (blue, green and red plots), are shown for two recording sites on the IFG and two sites on MFG. See Figure 3A for location of the sites. Lines and shaded areas represent mean high gamma ERBP and its standard error, respectively. Purple bars denote time windows where responses to the target stimuli were significantly larger than those to the same stimuli in the tones task (*q* < 0.01). Horizontal box plots denote the timing of behavioral responses to the target stimuli (medians, 10th, 25th, 75th, and 90th percentiles).

Task-related changes in high gamma activity were not restricted to the temporal lobe and were observed in IFG and MFG in both subjects with frontal lobe electrode coverage (Figure 5, Supplementary Figure 5). Targets elicited larger responses compared to when the same words were presented in a tone detection task in both IFG and MFG (purple bars in Figure 5). Minimal activity in both regions was observed in response to non-target speech stimuli when tones were targets, and phonemic and semantic processing were not necessary for task perfomance. In contrast, both targets and non-targets relevant to the task elicited responses in IFG in both subjects and MFG in subject L258 (red and green plots). Responses within MFG in subject L275 were restricted to target stimuli and had onset latencies longer than those observed at sites overlying either the superior temporal gyrus (STG), MTG or IFG, but were comparable to the timing of the late high gamma increases seen on posteromedial HG. These late increases in high gamma activity always preceded the subjects' behavioral responses (horizontal box plots in Figure 5 and Supplementary Figure 5), which elicited high gamma activity within both pre- and post-central gyrus (see Figures 3, 4).

### Differential response patterns to target stimuli: PLST vs. IFG

**Figure 6:**
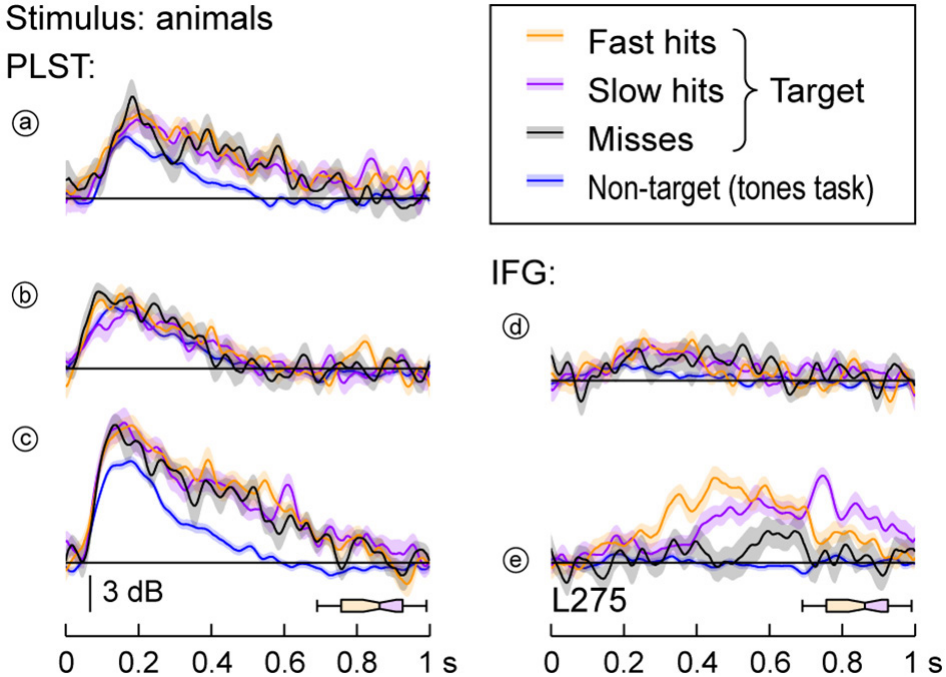
**High gamma responses to speech stimuli and the subject's behavioral performance**. Data recorded from sites (a to e) (see Figure 3A) in response to target animal stimuli are plotted separately for trials that were associated with fast behavioral responses (17 trials; orange), slow responses (17 trials; purple), misses (11 trials; black), and non-target trials from a tone detection task (200 trials; blue). Lines and shaded areas represent mean high gamma ERBP and its standard error. Horizontal box plots denote the timing of behavioral responses to the target stimuli (medians, 10th, 25th, 75th, and 90th percentiles). See Figure 3A for location of the sites.

Different response patterns elicited by target stimuli were noted between activity simultaneously recorded from PLST and IFG in subject L275. High gamma activity on PLST elicited by target stimuli (animals) did not significantly vary as a function of whether the subject responded rapidly or slowly or when the target was missed altogether (Figure 6, left column). In comparison, the same words when they were not relevant non-targets (tone detection task) elicited comparable early activity, but markedly diminished responses later in time [sites (a) and (c) in Figure 6]. In contrast to activity on PLST, activity within pars opercularis of IFG could be significantly modulated by the presence and timing of the behavioral response. This finding is exemplified at site (e) located on the dorsal portion of the pars opercularis (see Figure 6), where faster response times were associated with earlier peaks of activity when contrasted with slower behavioral responses. Additionally, misses were associated with markedly decreased responses compared to hits, and there was no response when the same stimulus was presented as a non-relevant, non-target during a tone detection task. For subject L258, parcelation of single-trial high gamma activity based on behavioral performance did not reveal consistent differences between PLST and IFG. This was due to highly variable responses and low response magnitudes, particularly in IFG.

## Discussion

### Posteromedial HG

As expected from previous studies, activity within posteromedial HG was highly sensitive to the acoustic characteristics of speech (e.g., Nourski et al., 2009; Steinschneider et al., 2013). In general, high gamma activity was greater for male talkers than female talkers. This finding reflects contribution from phase-locked responses to the lower fundamental frequency of male talkers relative to female talkers and was most prominently observed in the most posteromedial aspect of HG. This property is not unique to speech, as this region exhibits reliable phase-locked responses elicited by click trains at repetition rates of up to 200 Hz (Brugge et al., 2008, 2009; Nourski and Brugge, 2011). VOT was reflected in the timing of high gamma activity as a delay in the peak of high gamma response. This effect was most prominent in more central areas of HG, contrasting with the temporal representation of the voice fundamental. This apparent spatial differentiation may be a consequence of the tonotopic organization, wherein phase locking would most likely occur in high best frequency areas of the HG, whereas VOT would be represented in low frequency regions, due to the later onset of low frequency energy associated with voicing onset (Steinschneider et al., 1994). The absence of single and double-on responses previously reported (e.g., Steinschneider et al., 2013) can be attributed to the temporal smearing inherent to averaging of responses to unique and naturally-elicited speech exemplars characterized by different VOTs. Finally, responses reflecting differences in stop consonant POA were more subtle, and were likely a result of spectral smearing due to averaging of responses to 20 different exemplars of [cat] and [ten] across multiple talkers and the location of the recording sites with reference to the tonotopic organization of HG.

Activity within posteromedial and central HG was not strongly modulated by task requirements in all three subjects, and if it occurred (e.g., L275), it was later than task-related modulations in all other regions studied. Thus, current findings do not support the premise that human primary auditory cortex is the location where auditory object formation occurs. In contrast, studies in primary auditory cortex of experimental animals have shown robust responses reflecting auditory object formation, task-related activity, and reward expectancy (e.g., Fritz et al., 2003; Nelken and Bar-Yosef, 2008; Brosch et al., 2011; Niwa et al., 2012). The difference between the current observations and those in animals may reflect species differences and the relative complexity of auditory cortical organization in humans (Hackett, 2007). This complexity would be paralleled by greater functional specialization for primary and non-primary areas as the demands for vocal learning and auditory sequence learning become progressively more complex (Petkov and Jarvis, 2012).

Our findings in HG are consistent with several magnetoencephalograpgy and event-related potential (ERP) studies (Shahin et al., 2006; Gutschalk et al., 2008; Sabri et al., 2013; Simon, 2014; but see Bidet-Caulet et al., 2007). One study observed that during selective attention to one speech stream over another, the M100, but not M50 component of the neuromagnetic response, was modulated by the attended stream (Simon, 2014). This finding is consistent with our negative results, as the M50 component is dominated by generators in or near primary auditory cortex, while the M100 component reflects generators from multiple non-primary areas, particularly those in planum temporale (Liégeois-Chauvel et al., 1994). Another study sorted magnetoencephalograpy data according to whether or not target tones were detected in a multi-tone cloud background capable of producing informational masking of the targets (Gutschalk et al., 2008). Detected targets elicited an M100-like component that was not present when the target sounds were not detected. In contrast, both detected and undetected tones evoked auditory middle-latency and steady-state responses whose generators likely include prominent contributions from the primary auditory cortex on HG. It should be noted, however, that other studies utilizing auditory detection paradigms failed to find modulation of the N100 component (Shahin et al., 2006; Sabri et al., 2013). This negative result is not restricted to the auditory modality and has been observed in early cortical activity during visual target detection tasks (Bansal et al., 2014).

The minimal modulation of early high gamma activity that we observed replicates the findings in a previous intracranial study, where no effect was observed in the magnitude or timing of high gamma activity within posteromedial HG during a tone detection task relative to passive listening (Nourski et al., 2014a). Finally, functional neuroimaging studies have not shown consistent task-related changes in HG (Pugh et al., 1996; Leicht et al., 2010). When present, attention-related modulations occurred mainly in non-primary auditory cortex lateral to core areas (Petkov et al., 2004). This latter finding is consistent with task-related modulations currently seen in the most anterolateral portion of HG in one subject (see Figure 2). It must be acknowledged, however, that limited sampling inherent to human HG recordings may be responsible for the lack of consistent task-related effects seen in the three subjects studied here.

### PLST

Early activity on PLST, occurring within 200 ms after stimulus onset, was not strongly modulated by task requirements, mirroring a result seen in different subjects performing a tone detection task (Nourski et al., 2014a). Studies have demonstrated that early high gamma activity reflects more automatic processing that helps represent specific spectral characteristics of tone stimuli (Nourski et al., 2014a,b), as well as the remapping of acoustic speech characteristics to those representing phonetic categories (Chang et al., 2010; Travis et al., 2013; Mesgarani et al., 2014). In contrast, later high gamma activity on PLST could be strongly modulated by task requirements. Findings such as these are neither unique to humans nor restricted to the auditory system. For instance, during visual object detection tasks, single unit activity from neurons within areas V4 and IT of the monkey showed limited modulation as a function of the target stimulus in the initial response component, yet were strongly dependent on the specific target in later response segments (Chelazzi et al., 1998, 2001). The authors suggested that these later effects were based on feedback from higher visual centers involved in working memory, and reflected response bias toward the behaviorally relevant objects. A similar “top-down” mechanism that biases responses toward task-relevant stimuli may also be responsible for the currently observed effects in PLST.

Several studies have shown that neural patterns of activity in auditory cortex independently encode speaker identity and phonemic content of verbal speech (“Who” is saying “what”; e.g., Formisano et al., 2008; Mesgarani and Chang, 2012). We examined whether similar patterns independently encoding voice vs. speech content would emerge during the performance of the current target detection tasks, but found no clear differences. It should be noted, however, that in the study of Formisano et al. (2008), subjects passively listened to only three vowels spoken by three talkers. Here, subjects actively listened to 180 unique word exemplars spoken by an almost equal number of different talkers presented during semantic classification tasks and control conditions that included gender identification. Furthermore, the brain regions associated with gender identification were primarily located over the non-dominant right hemishere and distributed on the lateral portion of HG and Heschl's sulcus, as well as portions of the superior temporal sulcus (Formisano et al., 2008). The current study examined the dominant left hemisphere with limited sampling of HG, and did not sample neural activity in Heschl's sulsus or the superior temporal sulcus. In the study by Mesgarani and Chang (2012), the subjects were performing a different behavioral task (selective attention), and the neural activity only had to be capable of discriminating sentences spoken by two talkers (one male). It thus remains to be determined whether high gamma power, at least within PLST, is capable of independently determining multiple speaker identities (or gender) and phonemic content (e.g., Obleser and Eisner, 2009).

Response enhancement on PLST began prior to word offset during the semantic classification tasks (see Figure 6). The timing of response enhancement indicates that the effect was not driven by processes directly reflecting semantic classification, but instead represented the phonemic processing that must by necessity occur earlier in order to accurately decode the words. Further, the target words elicited a larger response than non-target words. As pointed out by Hon et al. (2009), any target enhancement that occurs within early sensory regions when a semantic target is detected must originate from higher-level brain areas providing relevant feedback to the lower areas. In the present study, subjects had been primed to know that the same two exemplar words for each semantic category would be presented in each successive recording block. This priming would allow subjects to know that, for instance, in the animals task, /d/ and /k/ would be the first phonemes in the target words ([dog] and [cat]) and thus provide additional information useful for the completion of the semantic task.

Response enhancement on PLST was also independent of task performance accuracy and reaction time. The same effect has been observed on PLST in a different subject performing a tone detection task, thus replicating current findings (Nourski et al., 2014a). Object-based detection tasks require two sequential processes, object formation followed by object selection (Shinn-Cunningham, 2008). The independence of the neural responses from behavioral measures are consistent with PLST being involved in the process of semantic object formation, yet not directly tied to the process of object selection. Similar observations have been made in the lateral belt field AL in macaque auditory cortex when performing a discrimination task using consonant-vowel syllables (Tsunada et al., 2011). In that study, single-cell responses reflected the categorization of the syllable (i.e., object formation), but did not vary as a function of the animal's behavioral performance (i.e., object selection). Activity that does not vary with behavioral performance likely reflects processes that precede sound object formation.

Even in the subject where later activity was strongly modulated (L275), effects were not uniform and showed site-by-site variability. This variability may partly explain why task-related modulation on PLST was not seen in subject L258 (see Supplementary Figure 5). Additionally, electrode array placement was more posterior along the STG in subject L258 when compared to the placement in L275. Electrical stimulation in subjects with epilepsy while they participated in various auditory and speech-related tasks has demonstrated the functional heterogeneity of the STG (Boatman, 2004), indicating that differences in electrode placement can be a major source of inter-subject variability. Finally, language processing skills of the subjects and effort necessary for successful performance of the task, may have also been a significant factor contributing to the inter-subject variability observed in this study.

### Auditory-related cortex

Multiple brain regions outside of the classically defined auditory cortex were differentially activated during the target detection tasks. For instance, task-related activity was shown within MTG, and enhancement of later activity was observed in responses to targets and non-targets in the semantic categorization tasks. Similar activation of MTG immediately adjacent to the superior temporal sulcus in response to speech has been reported (Figure 3 in Flinker et al., 2010). This region has been shown to be important in lexical processing, and is activated even during passive presentation of words (Dronkers et al., 2004; Indefrey and Cutler, 2004; Hagoort, 2005; Hickok, 2009). Unfortunately, sampling was too limited to better describe these modulations outside of observing that they had latencies comparable with those seen on PLST and frontal regions.

The IFG of the dominant left hemisphere was also activated during target detection tasks. High gamma activity was observed when stimuli were targets, and, to a lesser degree, non-targets. Findings are in keeping with other auditory target detection studies. Bilateral activation of the IFG occurred during an auditory detection task using positron emission tomography when targets were words, consonant-vowel syllables, or tone triplets (Fiez et al., 1995). Activation of the left IFG was observed in a study by Shahin et al. (2006) that combined functional MRI (fMRI) and ERP and used two target detection paradigms similar to that used in the current study: (1) a semantic task of detecting infrequent word targets denoting animals in a stream of words denoting non-animate objects; (2) a voice gender task detecting infrequent tokens spoken by males in a stream of words spoken by females. Results from fMRI were used to constrain possible anatomical source generators of the ERP. Activation of the IFG in the left hemisphere was seen in the semantic task performed with fMRI, and was associated with negative ERP components to both target and non-target words. Further, responses to targets were larger than responses to non-targets. Peak latencies of these negative ERP components were 450 and 600 ms, respectively, and overlap in time with the high gamma activity observed in the IFG in the present study. These results obtained from neurologically-normal subjects are all concordant with current results, despite the fact that all of our subjects were epileptic patients, and one subject (L275) was trilingual and had non-localizing cognitive deficiencies.

An important distinction between the responses located on PLST and IFG is that activity within pars opercularis of the IFG could vary as a function of behavioral performance (see Figure 6). Activity recorded during correctly identified targets was larger than when the target was missed. Further, activity during trials with shorter reaction times peaked earlier than activity during trials when reaction times were longer. This relationship with behavioral performance mirrors that seen in ventrolateral prefrontal cortex of macaques performing a phonemic discrimination task (Russ et al., 2008), and, as discussed above, contrasts with neural activity observed in field AL (Tsunada et al., 2011). The transformation in response characteristics from temporal to frontal lobe is parsimonious with the view that PLST is involved in the process of word object formation, while IFG is involved in the process of word object selection (Shinn-Cunningham, 2008).

MFG appears to also be involved in object selection, as it too responded only to targets (see Figure 5) and relevant non-targets during semantic categorization tasks (see Supplementary Figure 5). This activity began later than that in STG and IFG, yet preceded behavioral responses. Activation of the left MFG during a semantic target detection task has been reported using fMRI (Shahin et al., 2006). Variability in responses to targets and relevant non-targets has also been shown in detection tasks using visual stimuli (Kirino et al., 2000; Kiehl et al., 2001; Bledowski et al., 2004; Hampshire et al., 2007; Hon et al., 2012). To varying degrees, MFG as well as IFG were shown to respond either selectively to visual targets or to both targets and relevant non-targets. Additional work will be required to determine the sources of variability that characterized responses during the semantic classification tasks in IFG and MFG.

Strong task-related modulation of high gamma power outside classically defined auditory cortex is consistent with that seen in both the auditory and visual modalities in human ERP and fMRI studies (Sabri et al., 2013; Bansal et al., 2014). In the one study that compared responses to detected vs. undetected sound targets (Sabri et al., 2013), greater activation (as revealed by fMRI) was noted in the parietal lobe, thalamus and basal ganglia. While these regions were not examined in the current study, present results indicate that activity within IFG and MFG (as revealed by high gamma ERBP) is also related to the behavioral outcomes of the task, including the presence of the behavioral response and its timing.

## Concluding remarks

The response patterns described here reflect multiple processing stages of word object formation that constitute lexical encoding. At a neuroanatomical level, it does not appear that object formation occurs in posteromedial HG. Responses within this region are dominated by representation of the acoustic attributes of speech, and are therefore prelexical. Activity on PLST is also prelexical, but, in contrast to posteromedial HG, can also be strongly modulated by higher-order areas subserving lexical and semantic processing. The modulation on PLST during semantic classification tasks indicates that this region represents an early stage in word object formation.

It should be acknowledged that the subjects that participated in this study are patients who have neurologic deficits, including those in the language domain, and who have been treated with multiple anticonvulsant drugs over long periods of time. This calls into question as to whether findings in this population can be generalized to subjects without neurologic deficits. Despite this limitation, intracranial investigations of neurosurgical patients have been highly fruitful in defining organizational features of auditory and auditory-related cortex (e.g., Crone et al., 2001; Sahin et al., 2009; Chang et al., 2010; Mesgarani and Chang, 2012; Mesgarani et al., 2014). Findings described in the present report confirm and extend our own previous intracranial results demonstrating that PLST exhibits task-related modulation of high gamma activity regardless of behavioral outcome (Nourski et al., 2014a). Finally, results are congruent with non-invasive human studies (e.g., Pugh et al., 1996; Shahin et al., 2006; Gutschalk et al., 2008; Obleser and Eisner, 2009; Leaver and Rauschecker, 2010; Leicht et al., 2010; Simon, 2014) and relevant investigations using experimental animals (e.g., Russ et al., 2008; Brosch et al., 2011; Tsunada et al., 2011; David et al., 2012; Steinschneider et al., 2013; Sutter et al., 2013).

Future intracranial studies must corroborate current observations and extend them by examining task-related activity in other brain regions known to be important for sound processing. Specifically, investigation of response profiles in anterolateral HG, planum temporale, anterior STG, superior temporal sulcus and MTG will help identify additional stages of word object formation. Similarly, additional work will be needed to further characterize the roles of IFG and MFG in both dominant and non-dominant hemispheres in word object selection. Finally, future studies should include investigation of dynamic interactions between cortical regions, including feedback from higher-order cortices onto sensory areas. This will likely require examination of long-range phase coherence at multiple frequency bands (e.g., theta-gamma) that are likely important in long-range interactions between spatially disparate regions. As we continue investigation of these circuits, our conclusions will undoubtedly be refined and, hopefully, translationally relevant for the understanding of normal speech processing and its disfunction occurring in developmental language disorders, and acquired disorders such as stroke and normal aging.

### Conflict of interest statement

The authors declare that the research was conducted in the absence of any commercial or financial relationships that could be construed as a potential conflict of interest.

## Abbreviations

ECoG: electrocorticography
ERBP: event-related band power
ERP: event-related potential
fMRI: functional magnetic resonance imaging
HG: Heschl's gyrus
IFG: inferior frontal gyrus
MFG: middle frontal gyrus
MRI: magnetic resonance imaging
MTG: middle frontal gyrus
PLST: posterolateral superior temporal gyrus
POA: place of articulation
STG: superior temporal gyrus
VOT: voice onset time.
