# Supplementary material for: Differential activation of human core, non-core and auditory-related cortex during speech categorization tasks as revealed by intracranial recordings
Source: Front Neurosci. 2014 Aug 11;8:240. doi: 10.3389/fnins.2014.00240 (PMC4128221; doi:10.3389/fnins.2014.00240)
Supplement: Supplementary file 1 [file DataSheet1.DOCX]

***Supplementary Material***

**Differential activation of human core, non-core and auditory-related cortex during speech categorization tasks as revealed by intracranial recordings**

**Mitchell Steinschneider^1*‡^, Kirill V. Nourski^2‡^, Ariane E. Rhone^2^, Hiroto Kawasaki^2^, Hiroyuki Oya^2^,
Matthew A. Howard III^2^**

^1^Departments of Neurology and Neuroscience, Albert Einstein College of Medicine, Bronx, NY, USA

^2^Human Brain Research Laboratory, Department of Neurosurgery, The University of Iowa, Iowa City, IA, USA

***Correspondence:** Dr. Mitchell Steinschneider, Department of Neurology, Albert Einstein College of Medicine,
1300 Morris Park Ave., Bronx, NY, 10461, USA
E-mail: mitchell.steinschneider@einstein.yu.edu
Phone: +1 (718) 430-4115
Fax: +1 (718) 430-8588

**Supplementary Figures**


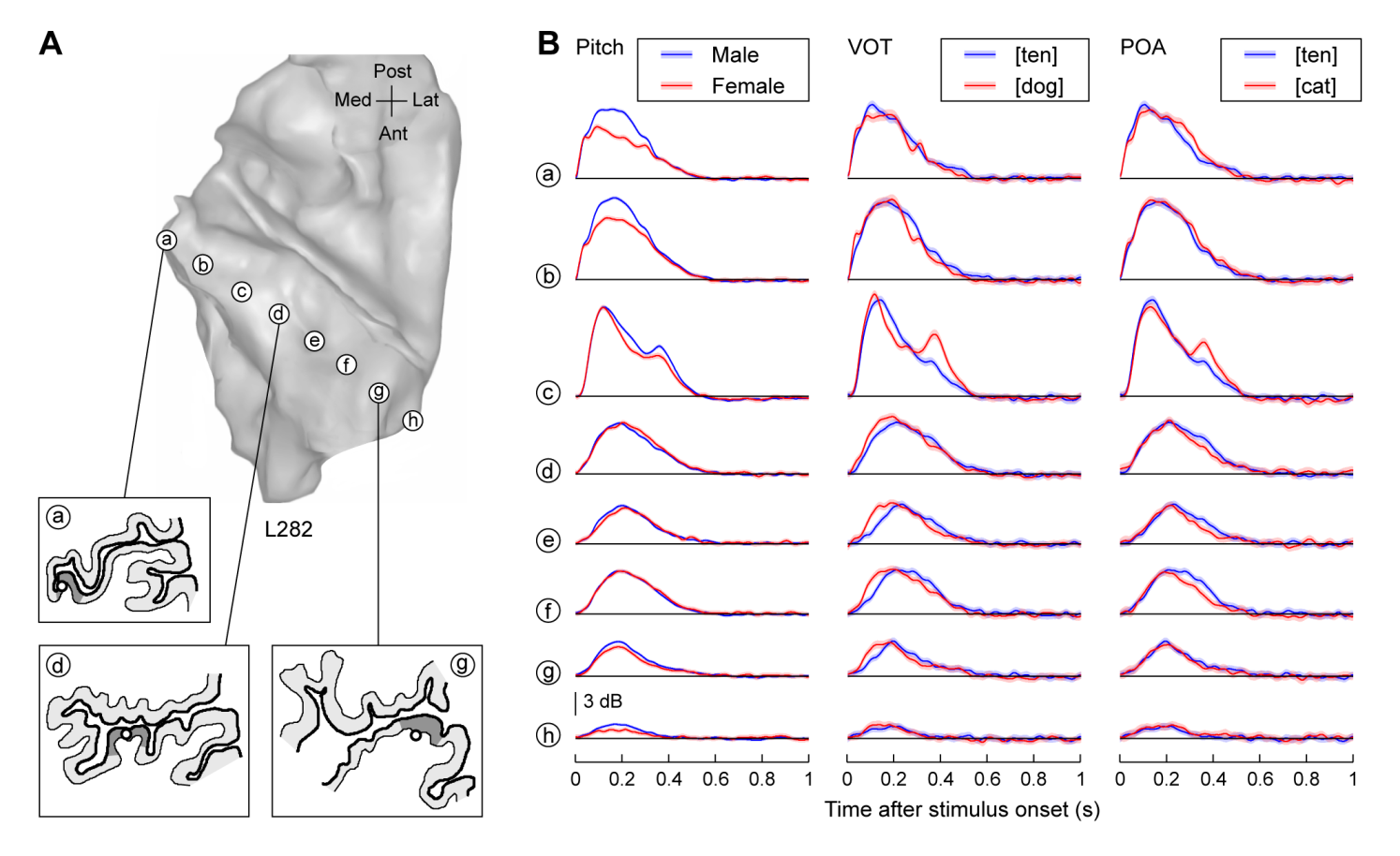


**Supplementary Figure 1. Representation of acoustic stimulus attributes in HG.** Confirmatory data from subject L282. (A): MRI of left superior temporal plane showing the locations of recording contacts chronically implanted in HG. Insets: tracings of MRI cross-sections showing the location of three recording contacts (circles) relative to the gray matter of the HG (dark gray shading). (B): High gamma responses to speech sounds differing in pitch, initial stop consonant VOT and POA are shown in the left, middle and right column, respectively. Lines and shaded areas represent mean high gamma ERBP and its standard error, respectively.


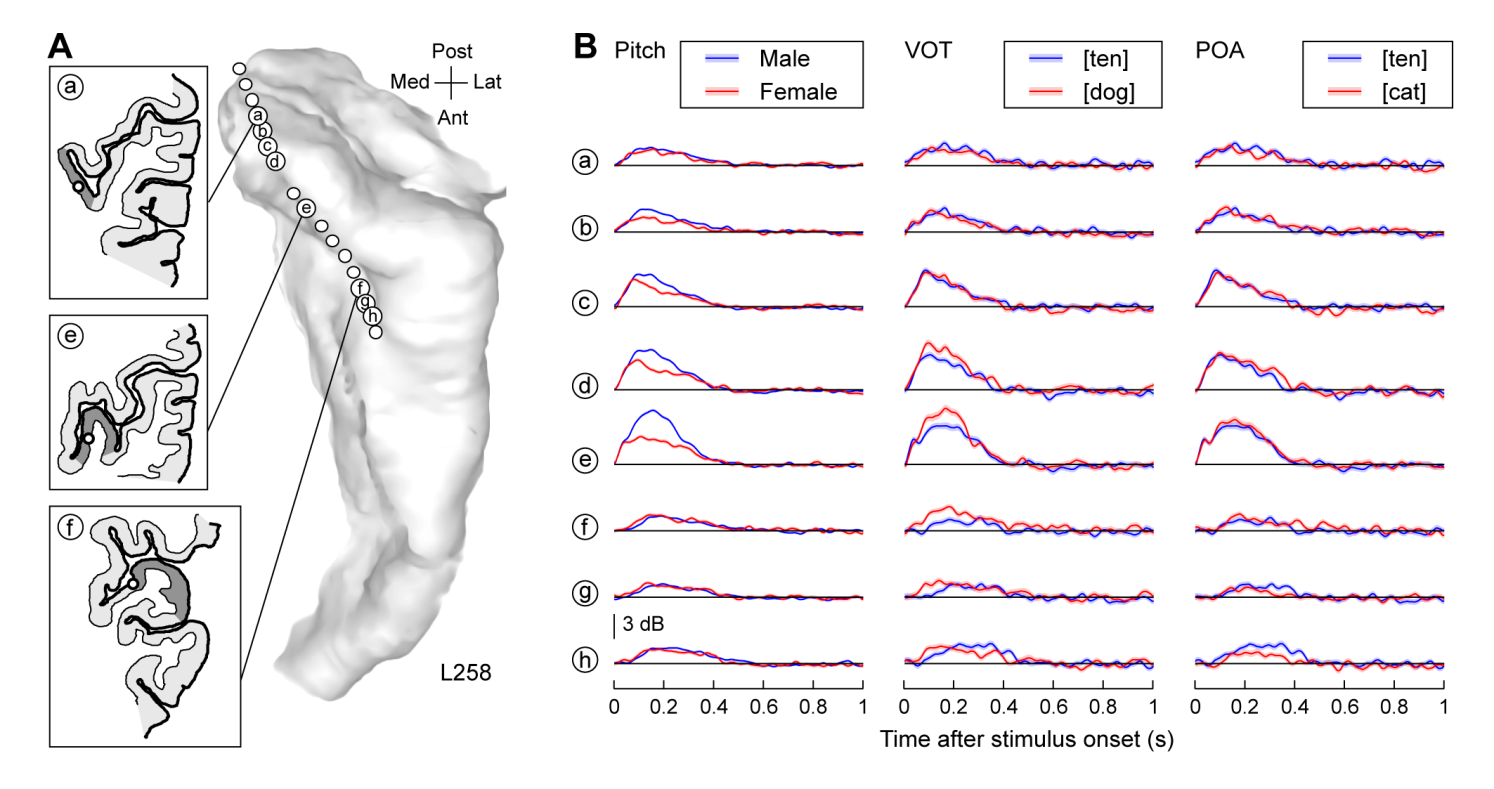


Supplementary Figure 2. Representation of acoustic stimulus attributes in HG. Confirmatory data from subject L258. (A): MRI of left superior temporal plane showing the locations of recording contacts chronically implanted in HG. Insets: tracings of MRI cross-sections showing the location of three recording contacts (circles) relative to the gray matter of the HG (dark gray shading). (B): High gamma responses to speech sounds differing in pitch, initial stop consonant VOT and POA are shown in the left, middle and right column, respectively. Lines and shaded areas represent mean high gamma ERBP and its standard error, respectively.


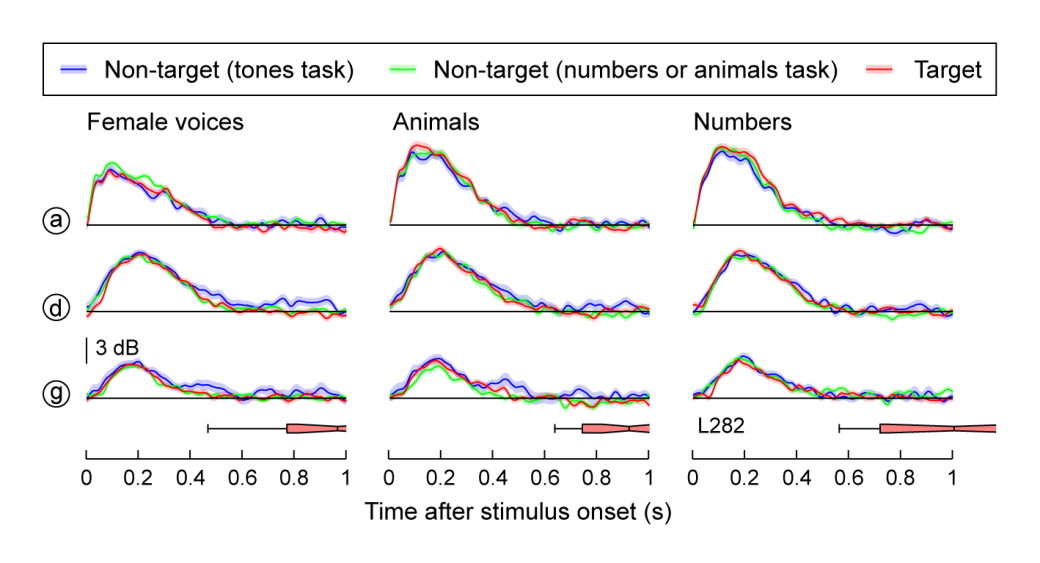


**Supplementary Figure 3.** **Task effects on responses to speech stimuli in HG.** Confirmatory data from subject L282. Responses to three types of stimuli (female voices, animals, numbers; left, middle and right column, respectively) are shown for three representative recording sites in HG (rows; see Supplementary Figure 1A for locations). Colors (blue, green and red) represent different task conditions. Lines and shaded areas represent mean high gamma ERBP and its standard error, respectively. Horizontal box plots denote the timing of behavioral responses to the target stimuli (medians, 10th, 25th, 75th and 90th percentiles).


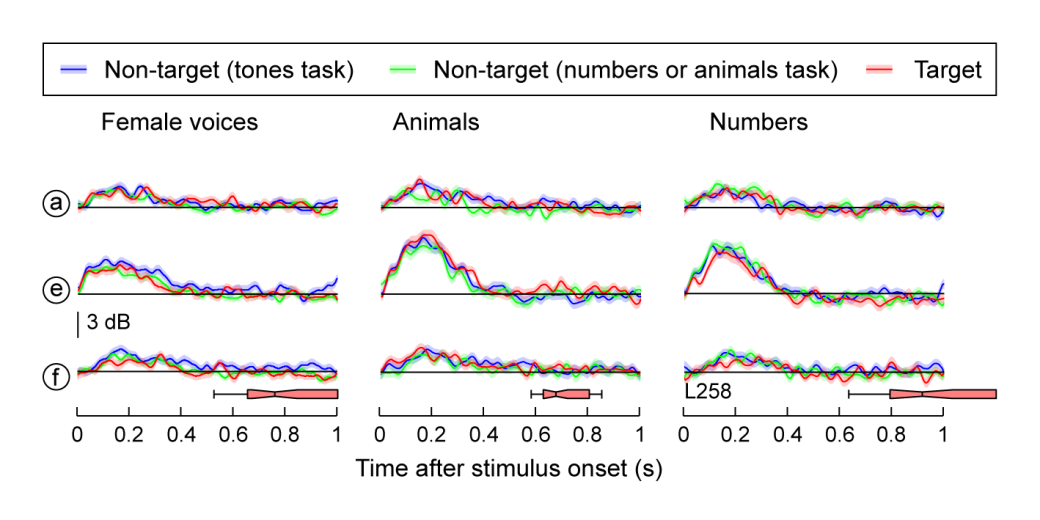


**Supplementary Figure 4.** **Task effects on responses to speech stimuli in HG.** Confirmatory data from subject L258. Responses to three types of stimuli (female voices, animals, numbers; left, middle and right column, respectively) are shown for three representative recording sites in HG (rows; see Supplementary Figure 1A for locations). Colors (blue, green and red) represent different task conditions. Lines and shaded areas represent mean high gamma ERBP and its standard error, respectively. Horizontal box plots denote the timing of behavioral responses to the target stimuli (medians, 10th, 25th, 75th and 90th percentiles).


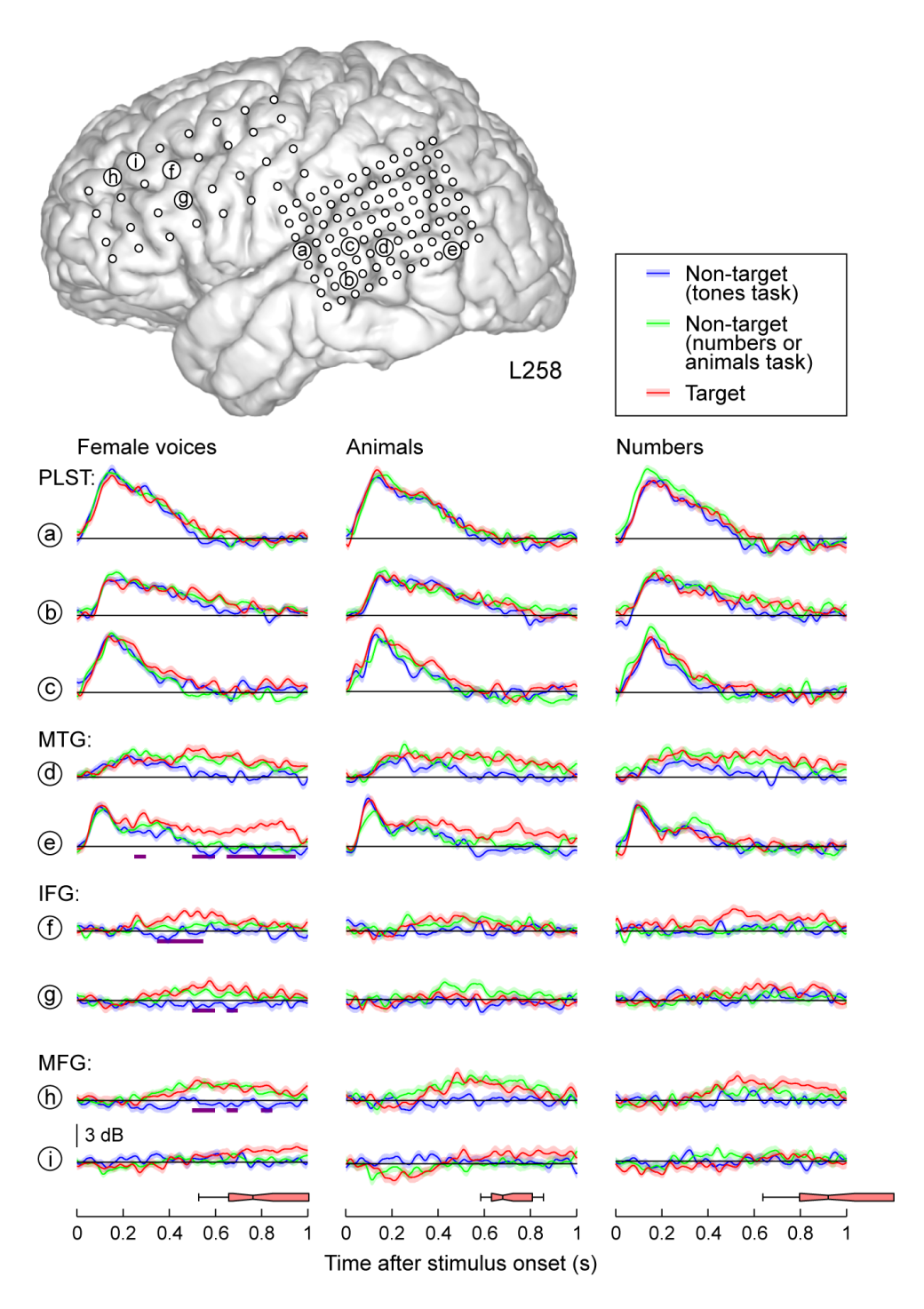


**Supplementary Figure 5. Task effects on responses to speech stimuli in PLST, MTG, IFG and MFG.** Data from subject L258. MRI of the left hemisphere showing the locations of chronically implanted subdural grid contacts and representative sites (a) through (i) is presented on the top. Responses to three types of stimuli (female voices, animals, numbers; left, middle and right column, respectively) are shown for representative recording sites (rows). Colors (blue, green and red) represent different task conditions. Lines and shaded areas represent mean high gamma ERBP and its standard error, respectively. Purple bars denote time windows where responses to the target stimuli were significantly larger than those to the same stimuli in the tones task (*q* < 0.01). Horizontal box plots denote the timing of behavioral responses to the target stimuli (medians, 10th, 25th, 75th and 90th percentiles).
